# A Conserved Female-Specific Requirement for the *GGT* Gene in Mosquito Larvae Facilitates RNAi-Mediated Sex Separation in Multiple Species of Disease Vector Mosquitoes

**DOI:** 10.3390/pathogens11020169

**Published:** 2022-01-27

**Authors:** Keshava Mysore, Longhua Sun, Ping Li, Joseph B. Roethele, Joi K. Misenti, John Kosmach, Jessica Igiede, Molly Duman-Scheel

**Affiliations:** 1 Department of Medical and Molecular Genetics, Indiana University School of Medicine, Raclin-Carmichael Hall, 1234 Notre Dame Ave., South Bend, IN 46617, USA; kmysore@iu.edu (K.M.); Longhua.Sun.15@nd.edu (L.S.); PLi@uams.edu (P.L.); jroethe@iu.edu (J.B.R.); jmisenti@iu.edu (J.K.M.); jkosmach@nd.edu (J.K.); jigiede@nd.edu (J.I.); 2 Eck Institute for Global Health, The University of Notre Dame, Notre Dame, IN 46556, USA

**Keywords:** *Aedes aegypti*, *Aedes albopictus*, *Anopheles gambiae*, *Culex pipiens*, *Culex quinquefasciatus*, development, female lethal, gamma-glutamyl transpeptidase, larvicide, male, RNAi, *Saccharomyces cerevisiae*, sex, yeast

## Abstract

Although several emerging mosquito control technologies are dependent on mass releases of adult males, methods of sex-sorting that can be implemented globally have not yet been established. RNAi screens led to the discovery of siRNA, which targets gamma-glutamyl transpeptidase (*GGT*), a gene which is well conserved in multiple species of mosquitoes and located at the sex-determining M locus region in *Aedes aegypti*. Silencing the *A. aegypti*, *Aedes albopictus*, *Anopheles gambiae, Culex pipiens,* and *Culex quinquefasciatus* *GGT* genes resulted in female larval death, with no significant impact on male survival. Generation of yeast strains that permitted affordable expression and oral delivery of shRNA corresponding to mosquito *GGT* genes facilitated larval target gene silencing and generated significantly increased 5 males:1 female adult ratios in each species. Yeast targeting a conserved sequence in *Culex GGT* genes was incorporated into a larval mass-rearing diet, permitting the generation of fit adult male *C. pipiens* and *C. quinquefasciatus*, two species for which labor-intensive manual sex separation had previously been utilized. The results of this study indicate that female-specific yeast-based RNAi larvicides may facilitate global implementation of population-based control strategies that require releases of sterile or genetically modified adult males, and that yeast RNAi strategies can be utilized in various species of mosquitoes that have progressed to different stages of sex chromosome evolution.

## 1. Introduction

Mosquito-borne diseases lead to hundreds of thousands of human deaths annually [1]. Although mosquito control is critical for prevention of mosquito-borne illnesses, the emergence of insecticide resistance, concern for the unwanted negative effects of insecticides on non-target organisms, and a general lack of support for mosquito control programs threaten current schemes for managing mosquitoes worldwide [2]. These problems have led to increased interest in alternative control methods, such as the sterile insect technique (SIT), which was proposed decades ago [3] and entails the release of sterile adult males with the goal of reducing large populations of insects. The broad implementation of SIT for control of mosquito populations has been hampered by a lack of cost-effective and scalable sex-sorting technologies that can be adapted for use worldwide in multiple mosquito species [4,5]. The use of males is critical for minimizing the health and nuisance biting risks posed by the release of females [4,5]. Likewise, the incompatible insect technique (IIT), which requires the release of *Wolbachia*-infected adult males for mosquito population suppression, can be paired with SIT and also involves mass-rearing and sex separation [6,7,8]. Transgenic-based population suppression approaches, including the release of insects which carry a dominant lethal (RIDL) [9,10], as well as several emerging gene drive technologies, also involve male mosquito releases [11,12]. Unfortunately, visual and mechanical separation technologies, the only existing sex-sorting methods for some species of mosquitoes, including *Culex pipiens* complex mosquitoes, are highly labor intensive and not sufficient for the large-scale implementation of SIT or IIT programs [5]. The establishment of sex separation methods is thus a rate-limiting step in the global deployment of several emerging population-based mosquito control technologies [5].

The identification and characterization of genes with sex-specific functions could promote the elucidation of male sex-sorting technologies to support emerging mosquito control interventions. In *A. aegypti*, sex determination is regulated by a non-recombining Y-chromosome-like male-determining region, referred to as the M locus, which is present on chromosome one [13,14] and contains the male-determining factor *Nix* [15]. *A. aegypti* males, which have one copy of the chromosome bearing the M locus and one which lacks it, have an M/m genotype. *A. aegypti* females, which lack the male-determining locus, have an m/m genotype [16]. Characterization and sequencing of the M/m locus had been obstructed by the presence of highly repetitive DNA at this centromeric region of chromosome one. However, recent innovations in sequencing technology generated an improved and re-annotated genome assembly [17,18] that enabled better estimation of the M/m locus. These efforts revealed the presence of many *long non-coding RNA (lncRNA*) genes, as well as several protein-encoding genes that are located at or tightly linked to the M/m locus [17], a location that is referred to herein as the M/m locus region.

Although clusters of loci that cause sex-specific lethal effects are proposed to reside within the M/m region [19,20], the identities of these genes were previously unknown. A recent RNA interference (RNAi) screen led to the identification of female-specific larval lethal genes located in the *A. aegypti* sex-determining region, including multiple *lncRNA* genes [21,22]. RNAi-based larvicides directed against several of the *lncRNA* genes kill female larvae during development; however, they have no impact on male mosquitoes [21]. Unfortunately, *lncRNA* genes have not yet been annotated in most mosquito species, and orthologs for these M/m locus region genes have not been described in other mosquitoes [21]. However, recent characterization of *A. aegypti MtnB,* a protein-encoding gene located in the M locus region that has known orthologs in other dipterans, indicated that a female-specific larval requirement of this gene is well-conserved among mosquitoes [22]. These results, which are interesting given that a sex-determining region on homomorphic chromosome 1 controls sex in *Aedes* and *Culex* mosquitoes, whereas *A. gambiae* has evolved heteromorphic X and Y chromosomes [23], support the hypothesis that a female-specific requirement for protein-encoding genes located at the *A. aegypti* M/m locus region is conserved among mosquitoes. Further evaluation of this hypothesis through examining the function of other M/m locus region genes is expected to provide insight into the evolution of mosquito sex chromosomes and sex-specific development.

Here, we pursue RNAi-mediated silencing of *gamma-glutamyl transpeptidase* (*GGT*), a gene located in the M/m locus region of *A. aegypti. GGT* is conserved in multiple species of dipteran insects, including disease vector mosquitoes. In *Drosophila melanogaster*, *GGT*, which is located on the X chromosome, is predicted to have glutathione hydrolase activity and peptidyltransferase activity [24]. *GGT* is expressed in a variety of *D. melanogaster* tissues, including the adult head, the embryonic and larval circulatory system, and midline ventral glial cells, in which it is proposed to function in the glutathione catabolic processes [24]. The human *GGT* gene has been implicated in glutathionuria, a disorder that is characterized by increased glutathione concentration in the plasma and urine [25]. Based on recent analyses of other M/m locus region genes in *A. aegypti* [21,22], it was hypothesized that silencing *GGT* during mosquito larval development will result in significantly higher male:female sex ratios in multiple mosquito species, and that the development of scaled RNAi approaches for targeting *GGT* could facilitate the mass-rearing of male mosquitoes.

*Saccharomyces cerevisiae*, which is genetically tractable and inexpensive to produce at scale, can be engineered to produce interfering RNA that silences mosquito genes upon consumption of the yeast by larvae [26,27,28]. Yeast RNAi strains promote effective gene silencing during the larval stages, and yeast RNAi larvicides generate relatively higher levels of larval mortality than soaking or chitosan RNAi larvicide delivery methods do [26,27]. Moreover, RNA can be generated through yeast culturing, significantly reducing interfering RNA production costs [28]. The interfering RNA also retains activity when the yeast is heat-killed and dried, which allows it to be more readily packaged, shipped, stored, and used in an inactivated form [26,27]. Initial studies in *A. aegypti* suggest that yeast RNAi silencing of M/m locus region genes permits the scaled production of fit adult males [21,22]. Here, we extend the use of this yeast system to the characterization of mosquito *GGT* orthologs, as well as to the scaled production of male *Culex pipiens* complex mosquitoes.

## 2. Results

### 2.1. RNAi Assays Identify GGT as a Female-Specific Larval Lethal Gene in A. aegypti

**Figure 1:**
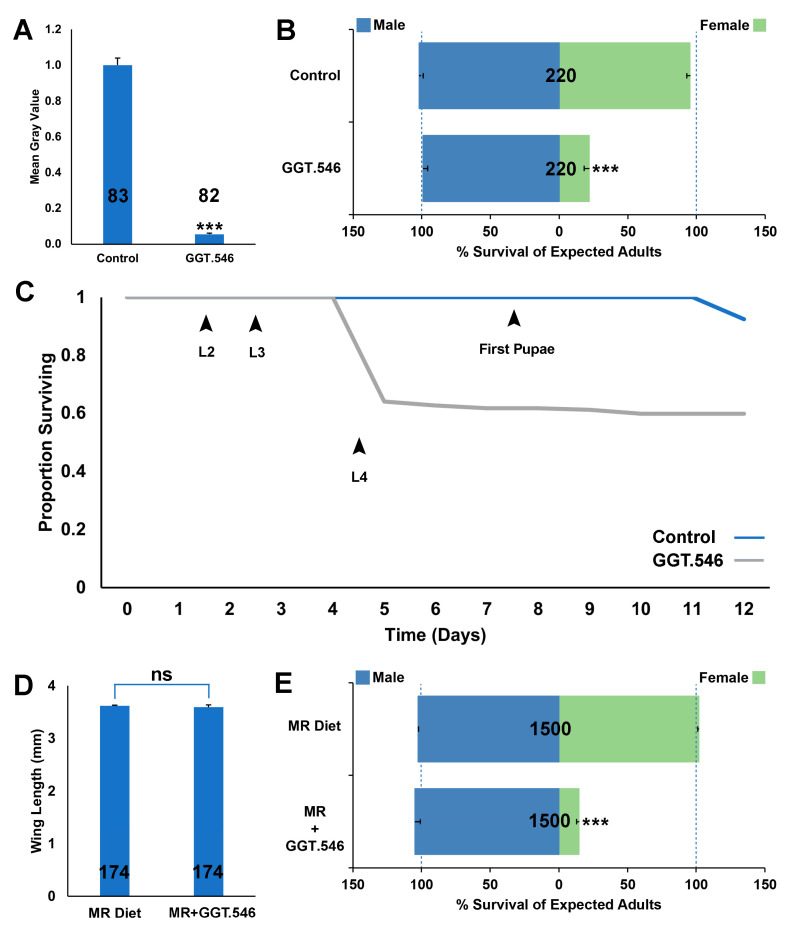
Yeast larvicide GGT.546 functions as a female-specific larvicide in *Aedes aegypti.* (**A**) Silencing of *GGT* in *A. aegypti* following larval consumption of GGT.546 yeast was verified through mean grey value quantification of *GGT* transcripts following in situ hybridization experiments performed on L4 brain tissue (*** = *p* < 0.001, Student’s *t*-test; error bars denote standard deviation). Larval consumption of GGT.546 yeast resulted in significant female larval mortality (**B**), which occurred primarily in the third instar (GGT.546-treated larvae = gray line in survival curve shown in (**C**); compared with the blue line, which denotes the survival of control-interfering RNA yeast-treated larvae), yet did not significantly impact survival of males through adulthood (*p* > 0.05, chi-squared test). (**D**) No significant differences in mean wing lengths were detected in adult males reared following scaled rearing of larvae on a mass-rearing diet prepared with GGT.546 yeast (MR+GGT.546; *p* > 0.05 vs. wings from males reared on the standard mass-rearing diet (MR Diet), which served as a control, *t*-test). Although the consumption of MR+GGT.546 resulted in significant female mortality, it had no impact on male survival (**E**). The results shown in panels (**B**,**C**) correspond to container trials performed on 20 larvae per replicate container; results from larvae that had consumed control-interfering RNA yeast, which had no impact on male or female survival (*p* > 0.05, chi-squared test) are shown for comparison. Results shown in (**D**,**E**) correspond to trials performed in scaled rearing trays containing 500 larvae that consumed the MR diet or the MR+GGT.546 diet; data are shown as mean survival through adulthood; *** = *p* < 0.001, chi-squared test. Throughout this figure, the N numbers for each treatment are indicated on the respective columns of each bar graph, and error bars represent standard errors of the mean.

Given evidence that the *A. aegypti* M/m locus is tightly linked to developmental genes that confer sex-specific effects [21,22,29], it was hypothesized that the *A. aegypti* GGT gene, which is located on Chromosome 1 and flanks the sex determination M/m locus [17,18], functions as a sex-specific lethal gene. In support of this hypothesis, larval soaking assays performed with siRNA #546, which corresponds to the GGT transcript, induced significant larval mortality, resulting in 25 ± 3% of expected adult females (*p* < 0.001), but had no significant impact on male survival. Based on the outcome of these trials, yeast strain GGT.546, which expresses an shRNA corresponding to the siRNA #546 target site in GGT, was constructed and found to induce 94 ± 1% reduction in *GGT* transcript levels during larval development (Figure 1A, *p* < 0.001 in comparison with larvae reared on control-interfering RNA yeast, which expresses an shRNA hairpin with no known target in mosquitoes, and which is referred to as control-interfering RNA yeast hereafter). In small container trials performed on 20 larvae, treatments with GGT.546 throughout larval development induced significant larval death (Figure 1B, *p* < 0.001), yielding only 22 ± 4% of expected females, yet had no significant impact on male survival (Figure 1B, *p* > 0.05), resulting in significantly higher male:female ratios among surviving adult mosquitoes (Figure 1B, *p* < 0.001). Larval death occurred primarily during the third larval instar, as shown in the survival curve in Figure 1C.

GGT.546 larvicide was also evaluated when 500 larvae were reared in larger rearing trays. In these scaled rearing experiments, GGT.546 yeast was incorporated into a larval mass-rearing diet (Figure 1E). Significant larval mortality resulted when larvae consumed this diet, yielding 15 ± 1 of expected adult female survival. There was no significant impact on male survival (Figure 1F) or wing length, a correlate of fitness (though fitness was not measured directly, Figure 1D), resulting in significantly higher male:female ratios among surviving adult mosquitoes (Figure 1E, *p* < 0.001).

### 2.2. The Female-Specific Larval Requirement for GGT Is Conserved in Multiple Species of Disease Vector Mosquitoes

*GGT* orthologs have been identified in the genomes of *A. albopictus, C. pipiens*, *C. quinquefasciatus*, and *A. gambiae* (Table 1). siRNAs #565 (which corresponds to *A. albopictus GGT*), #560 (which targets *A. gambiae GGT*), and #566 (which corresponds to both the *C. quinquefasciatus* and *C. pipiens GGT* genes) induced significant female larval death, yet did not affect male survival following soaking treatments of each respective species (detailed results and statistics are provided in Table 1). Based on these results, as well as those observed in *A. aegypti* (Figure 1, Table 1), yeast strains which express shRNA corresponding to either the #560, #565, or #566 target sites, hereafter referred to as GGT.560, GGT.565, and GGT.566, were thus constructed. shRNA expression was verified in each of these strains (Figure S1).

**Figure 2:**
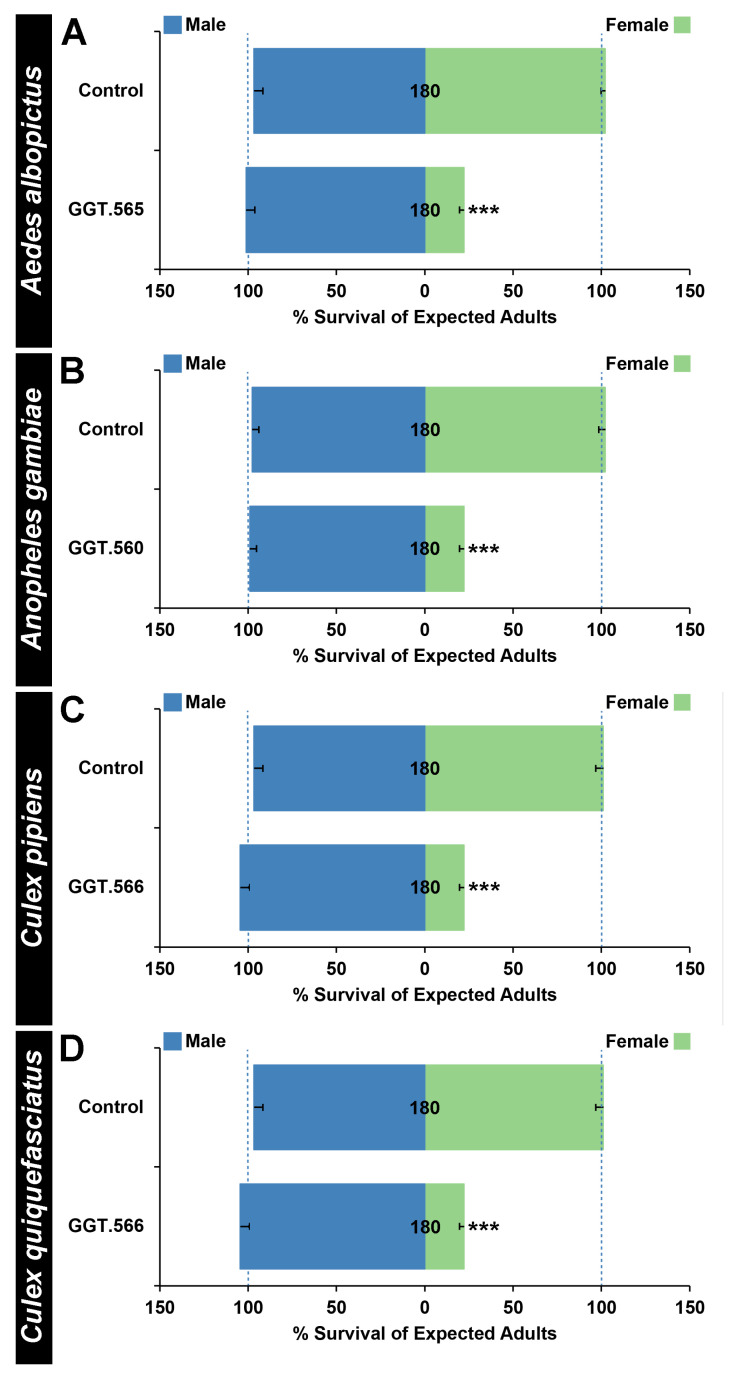
A female larval requirement for GGT function in multiple species of mosquitoes. Larval consumption of yeast-interfering RNA larvicides corresponding to the *A. albopictus* (GGT.566 in (**A**)), *A. gambiae* (GGT.560 in (**B**)), *C. pipiens* (GGT.566 in (**C**)), and *C. quinquefasciatus* (GGT.566 in (**D**)) *GGT* genes results in significant female-specific mortality in each species (*** = *p* < 0.001, chi-squared test), but does not impact male survival ((**A**–**D**), *p* > 0.05, chi-squared test). Rearing larvae on control-interfering RNA yeast, which lacks a known target in mosquitoes, had no significant impact on female or male survival ((**A**–**D**), *p* > 0.05, chi-squared test). Data are displayed as mean survival through adulthood, with error bars corresponding to SEM. The N numbers corresponding to each treatment are indicated on the respective columns of each bar graph, and error bars represent standard errors of the mean.

Rearing *A. albopictus* larvae on GGT.566 yeast resulted in significantly higher than expected male:female ratios (Figure 2A, *p* < 0.001). Although only 22 ± 3% of expected *A. albopictus* female mosquitoes treated with GGT.566 emerged as adults (Figure 2A, *p* < 0.001), no significant impact on male survival was observed (Figure 2A, *p* > 0.05). Likewise, although GGT.560 had no significant impact on the survival of males through adulthood (Figure 2B, *p* > 0.05), only 22 ± 2% of expected *A. gambiae* female mosquitoes survived through adulthood (Figure 2B, *p* < 0.001), resulting in a significantly higher male:female ratio among adult survivors (Figure 2B, *p* < 0.001). Finally, significantly higher than expected male:female ratios were also observed among both *C. pipiens* (Figure 2C, *p* < 0.001), and *C. quinquefasciatus* (Figure 2D, *p* < 0.001) adult survivors following larval rearing on GGT.566 yeast, a difference that coincided with 22 ± 3% of expected *C. pipiens* (Figure 2C, *p* < 0.001), and 22 ± 3% of expected *C. quinquefasciatus* (Figure 2D, *p* < 0.001) female adult emergence, yet had no impact on male survival of either species (Figure 2C,D, *p* > 0.05).

### 2.3. The Use of GGT.566 Yeast in Scaled Production of Adult C. pipiens Complex Male Mosquitoes

**Figure 3:**
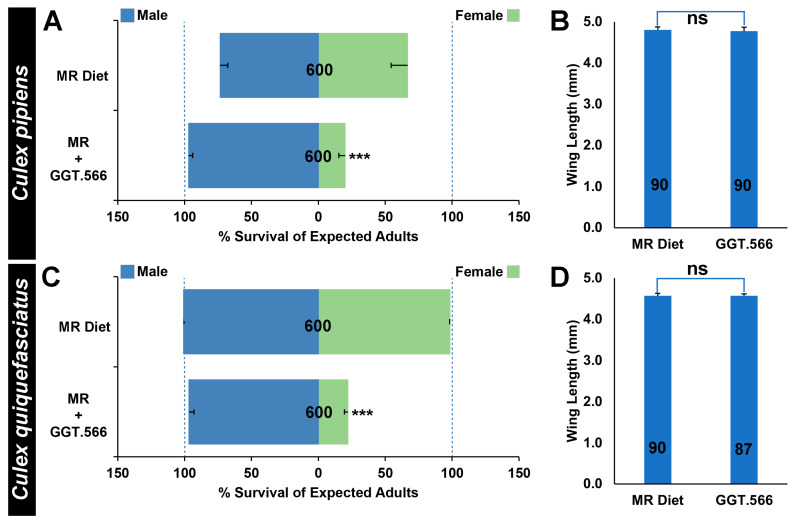
GGT.566 yeast facilitates scaled production of fit *C. pipiens* complex males. Incorporation of GGT.566 yeast into a *Culex* larval mass-rearing diet (MR+GGT.566) induced significant female *C. pipiens* ((**A**), *** = *p* < 0.001, chi-squared test) and *C. quinquefasciatus* ((**C**), *** = *p* < 0.001, chi-squared test) mortality, yet did not significantly impact male survival ((**A**,**C**) *p* > 0.05, chi-squared test) or wing size (*C. pipiens* male wing results are shown in (**B**) and *C. quinquefasciatus* male wing results are shown in (**D**); *p* > 0.05 vs. mass-rearing diet results for both sets of wing size data in (**C**,**D**), Student’s *t*-test). Data are displayed as mean survival through adulthood in (**A**,**C**) and mean adult male wing size in (**B**,**D**). Mosquitoes reared on the standard MR diet served as controls in all of these experiments (**A**–**D**). The N numbers corresponding to each treatment are indicated on the respective columns of each bar graph, and error bars represent standard errors of the mean. ns: no difference.

To assess whether the RNAi-based yeast larvicides could also facilitate scaled production of *C. pipiens* complex male mosquitoes, a larval diet and feeding regimen employed at *Aedes* mass-rearing facilities [30] was modified through replacement of the nutritional yeast component of the diet with dried inactivated yeast larvicide GGT.566, as well as through the addition of Koi food, a typical component of the *Culex* larval laboratory diet. The modified diet was tested on both *C. pipiens* (Figure 3A) and *C. quiquefasciatus* (Figure 3C) larvae grown in scaled larval rearing trays. Under these conditions, the larvicides continued to induce significantly higher than expected *C. pipiens* male:female ratios (Figure 3A, *p* < 0.001) resulting from significant female-specific mortality (Figure 3A, *p* < 0.001); however, there was no significant impact on male *C. pipiens* survival (Figure 3A, *p* > 0.05) or wing lengths (Figure 3B, *p* > 0.05), which correlate with fitness. Similarly, the modified diet permitted scaled rearing of fit *C. quinquefasciatus* males, with GGT.566 diet treatments inducing significantly higher male:female ratios (Figure 3C, *p* < 0.001) resulting from significant female-specific mortality (Figure 3C, *p* < 0.001), but no significant impact on male *C. quinquefasciatus* survival (Figure 3C, *p* < 0.001) or fitness, which was not measured directly but estimated through measurement of wing lengths, which were not significantly different (Figure 3D, *p* > 0.05).

## 3. Discussion

### 3.1. Sex Separation through Female-Specific Yeast RNA Larvicides

RNAi-based larvicides [28,31] and adulticides [32,33] are presently being assessed and may one day be incorporated into integrated mosquito control programs. Here, we provide evidence that RNAi-based insecticides could also facilitate mosquito sex separation, a requirement for SIT, IIT, as well as several genetic-based mosquito control technologies that depend on the mass release of male mosquitoes. Papathanos et al. [34] recommend that sex-sorting methodology should be stable, simple, and lead to the isolation of competitive males, criteria that are fulfilled by the yeast system described here (Figure 1, Figure 2 and Figure 3), which is also amenable to cost-effective and scalable production of shelf-stable yeast which can be dried and distributed for use in resource-limited regions of the world [28,31]. Moreover, yeast sex sorting systems, which are based on conditional gene silencing, induce no permanent changes to the mosquito genome, bypassing the need to transgenically manipulate existing mosquito strains that have been developed for population-based mosquito control strategies. Furthermore, the yeast would be heat-killed prior to use indoors at mass-rearing facilities, requiring no release of live GMOs into the environment. Thus, the *GGT*-silencing female-specific yeast-interfering RNA strains described herein, as well as the *lncRNA*- and *MtnB*-silencing strains recently described [21,22], satisfy many of the desired criteria for an effective sex-sorting system [34]. These findings suggest that future efforts to scale yeast production [28] for distribution to mass-rearing mosquito facilities will be useful.

Here, we demonstrated that replacement of the nutritional yeast component of an *A. aegypti* larval mass-rearing diet [30] with yeast that silences *GGT* resulted in the production of male mosquitoes at a five-fold higher number than females (Figure 1E). These results were similar to those described for yeast strains that silence *MtnB* [22] or *lncRNA* [21] genes, except that the GGT.546 yeast strain induces female-specific death during the third instar, slightly earlier than the other strains, which killed fourth-instar larvae. Given that earlier death of females is expected to reduce rearing costs [34], use of the GGT.546 strain may offer an advantage over the other yeast strains [21,22]. Moreover, use of the GGT.560 strain for targeting *A. gambiae* (Figure 2B) yielded higher levels of female mortality than did the MtnB.523 yeast strain [22], (78 ± 3% vs. 43 ± 5%, respectively), suggesting that this strain may be a better choice for the separation of *A. gambiae.* The availability of multiple strains leads to the question of whether mixing yeast strains could lead to higher levels of female mortality. Preliminary data in which multiple types of yeast were simultaneously fed to the mosquito larvae suggest that this is not the case. However, a strain that produces high levels of multiple shRNAs has yet to be constructed, and engineering such a strain may prove useful.

Given that none of the strains generated to date have resulted in the elimination of all females (Figure 1, Figure 2 and Figure 3; [21,22]), the present RNAi-based yeast systems will not be sufficient as a stand-alone strategy for mass male mosquito production. In addition to exploring methods of producing higher levels of one or more shRNAs, it could also be helpful to combine yeast technology with other existing sex separation methods that have been developed. A total of >99% male separation serves as the present standard, and this standard has been achieved in *A. aegypti* through the use of a sophisticated camera system [8], as well as through fluorescent-based sorting of mosquito larvae using complex parametric analyzer and sorter (COPAS-) flow cytometry for sex separation in several species [4,35,36]. Although these systems are extremely effective, it is not yet clear if this automated technology can be adapted for use in all disease vector mosquito species or readily deployed worldwide, particularly to locations with limited resources. *Aedes* and *Culex* mosquitoes display sexually dimorphic size and growth rates, with female pupae developing more slowly and being larger in size, facilitating the early physical removal of small pupae. Likewise, pupae display differences in terminal abdominal segments that facilitate visual separation [5]. Replacing nutritional yeast with larvicidal yeast during the rearing process could serve to reduce the labor-intensive nature of manual separation strategies. Furthermore, combination of yeast larvicide-based sorting with behavioral sorting techniques could also prove useful. For example, females can be drawn to host cues, permitting separation [5]. Combining yeast larvicide use with these types of separation techniques could facilitate broadened use of emerging population-based mosquito control strategies.

### 3.2. Scaled Rearing Culex pipiens Complex Males

*Culex pipiens* complex mosquitoes, which transmit both arboviruses, such as West Nile virus, and parasites that cause lymphatic filariasis, pose significant threats to human health [1]. These mosquitoes also present severe threats to birds located on islands throughout the tropics, where avifauna are particularly susceptible to population declines and extinctions resulting from the introduction of non-native pathogens transmitted by *Culex* mosquitoes. The development of SIT, IIT, and transgenic-based strategies for population-based *Culex* mosquito control on islands would be useful, but the development of such tools has been somewhat delayed with respect to comparable work in *A. aegypti* and *A. gambiae.* A relative lack of focus on *Culex* has resulted in the need for improved culturing methods, advances in genetic modification technology, *Wolbachia* transinfections, and further development and expansion of SIT programs, and as the development of population-based *Culex* control technologies progresses, the need for sex separation methodologies to facilitate these procedures is growing [4,5]. Female-specific yeast larvicide technology could thus particularly benefit the development and implementation of population-based control strategies for *Culex* (Figure 3)*,* for which sex-sorting methods outside of visual and physical separation have yet to be established [4,5].

Previous work demonstrated that silencing the *C. quinquefasciatus MtnB* gene resulted in female-specific larval death [22], but the target site for the yeast-interfering RNA larvicide used to target this gene was not conserved in *C. pipiens.* The GGT.566 target site is conserved in both species, and successful incorporation of yeast larvicide GGT.566 into a *Culex* rearing diet led to significant female mortality in both *Culex* species (Figure 3A,C). It is possible that this site could be conserved in other species of *Culex* vector mosquitoes, which would permit extension of this technology to other vectors. This could be particularly beneficial to regions where it is anticipated that multiple species of *Culex* mosquitoes may be present, which may complicate population-based control efforts. Although 97% survival was observed in GGT.566-treated *C. pipiens* males, only 67% *C. pipiens* male survival was observed when larvae were reared on the unmodified standard mass-rearing diet used in this study (Figure 3A). It is likely that GGT.566-treated male larvae in larvicide-treated containers experienced less competition for food (due to female larval death) and also ate dead female larvae in addition to the food supplied. The eating of dead larvae, which we often observe in our yeast larvicide-treated containers, could perhaps serve to improve male larval nutrition and survival in rearing trays with high larval densities, a potential added benefit for mass-rearing facilities if yeast RNAi sex separation technology were to be employed. This could be further explored. Moreover, our results suggest that the *Culex* diet used in the present study could stand to be further optimized.

### 3.3. Female-Specific Lethal Larvicides: Implications for Mosquito Sex Chromosome Evolution

The findings reported herein could have implications for the study of sex chromosome evolution [37,38,39], which is believed to initiate with a homologous pair of autosomes that acquire sex-determining loci, eventually leading to a proto-Y chromosome with a male fertility locus (M), as well as a proto-X chromosome carrying a male sterility locus (m). Suppressed recombination in the sex-determining region develops and eventually spreads along the proto-sex chromosomes, which evolve into separate heteromorphic X and Y chromosomes. Unlike *A. gambiae*, which has evolved a Y chromosome with the male-determining *Yob* gene [29], Culicines have retained homomorphic sex chromosomes, though a sex-differentiated region of suppressed recombination is believed to extend approximately 100 Mb beyond the sex determination M/m locus in *A. aegypti* [15,17,40,41,42,43]. Despite the different rates of sex chromosome evolution in mosquitoes, this investigation functionally verified that *GGT* is required for survival of female larvae in *A. aegypti* (Figure 1), *A. albopictus* (Figure 2A), *A. gambiae* (Figure 2B), *C. pipiens* (Figure 2C and Figure 3A), and *C. quinquefasciatus* (Figure 2D and Figure 3C). These findings, combined with a recent study that identified a conserved female-specific larval requirement for *MtnB* in mosquitoes [22], suggest that loci which cause sex-specific lethal effects that shape the boundaries of the sex-determination locus had begun to accumulate on the sex chromosomes prior to separation of the *Culicine* and *Anopheline* lineages.

Krzywinska et al. [20] described sex-specific lethality in *A. aegypti* that was associated with the inheritance of sex chromosomes produced through male meiotic recombination events that had occurred between the sex-determination locus and a linked EGFP-positive transgene, which served as a marker. They posed several explanations for this, and one that is consistent with both the *MtnB* study [22], as well as the *GGT* silencing results presented here, suggested that such recombination events might result in loss of a haploinsufficient sex chromosome gene for which m/m females require two copies. If *GGT*, as well as *MtnB* [22], M/m locus region *lncRNA* genes [21], and perhaps other genes in the M/m region are haploinsuffient in *A. aegypti* females, any female that inherits a recombinant m chromosome lacking a copy of any of these genes will die, a phenomenon that is sometimes referred to as ‘suppression of recombination’ due to the lack of resulting female offspring in the adult population. Thus, the early presence of haploinsufficient genes adjacent to the sex determination locus—prior to separation of the Culicines and Anophelines—may have helped to shape the boundaries of the sex determination region during mosquito sex chromosome evolution. Given these findings, it will be interesting to further assess this haploinsufficiency hypothesis and to assess the molecular mechanisms that might underlie it.

## 4. Materials and Methods

### 4.1. Mosquito Strains and Rearing

Mosquitoes were reared as described [22,44], except that an artificial membrane feeding system (Hemotek Limited, Blackburn, UK) was used to blood-feed adult females with sheep blood that had been purchased from a commercial vendor (HemoStat Laboratories, Dixon, CA, USA). The following strains of mosquitoes were used in these studies: *A. aegypti* Liverpool-IB12 strain, *A. albopictus* Gainesville strain (BEI Resources, NIAID, NIH: *A. albopictus* MRA-804, contributed by Sandra A. Allan), *A. gambiae* G3 strain (BEI Resources, NIAID, NIH: MRA-112, contributed by Mark Q. Benedict), and *C. quinquefasciatus* Strain JHB (provided by the Centers for Disease Control and Prevention for distribution by BEI Resources, NIAID, NIH: NR-43025). The *C. pipiens* strain was established from ovitrap collections in Niles, Michigan, with approximately 10th generation larvae used in these studies.

### 4.2. Larval siRNA Soaking Experiments

The Integrated DNA Technologies (IDT) Dicer-Substrate siRNA (DsiRNA) tool [45] was used to identify small interfering RNAs (siRNAs) corresponding to mosquito *GGT* genes (Table 1). These custom siRNAs (see Table 1), as well as a control siRNA with no known mosquito target [46], were purchased from IDT (Coralville, IA, USA) and used in larval soaking experiments. These assays, which were performed in duplicate as described [47], were executed using 20 first-instar larvae (L1) soaked in 0.5 μg/μL siRNA (Table 1) for 4 h and subsequently reared as described [26]. The chi-squared test was used to identify statistically significant differences between the observed and expected survival of adult female and male mosquitoes; a 1 female: 1 male ratio was expected on the basis of extensive control experiments performed in the insectary with the mosquito strains indicated above.

### 4.3. Production of Yeast RNAi Larvicides

RNAi yeast strains for silencing mosquito *GGT* genes were generated as described [27] by cloning of custom shRNA expression cassettes (synthesized by Invitrogen, Carlsbad, CA, USA) corresponding to *GGT* target gene sequences of interest (Table 1). The shRNA expression cassettes were inserted into the *pRS426 GPD* shuttle vector [48] and used to transform *S. cerevisiae CEN.PK* yeast (genotype *MATa/α ura3-52/ura3-52 trp1-289/trp1-289 leu2-3_112/leu2-3_112 his3 Δ1/his3 Δ1 MAL2-8C/MAL2-8C SUC2/SUC2* [49]. Dried inactivated yeast-interfering RNA larvicide was prepared as previously detailed [50] following confirmation of shRNA expression via PCR amplification, which was performed as described [33] using the following primers: forward 5′-TGCCGTAAGCATGTTTGAATGCTTC-3′ (specific to the 3′ side of the *A. aegypti* GGT.546 hairpin), forward 5′-GACGGTAAACTGGTCAAACAGATAG-3′ (specific to the 3′ side of the *A. gambiae* GGT.560 hairpin), forward 5′-TAAAACTAAGTAGTATCCCACTTGC-3′ (specific to the 3′ side of the *A. albopictus* GGT.565 hairpin), forward 5′-TACTGTTTTCATCAATCGTAAGTCT-3′ (specific to the 3′ side of the *C. quinquefasciatus* GGT.566 hairpin), forward 5′-ACGCTAACATCTATCAGTGC-3′ (specific to the 3′ side of the control hairpin, as previously described [33]) in combination with the reverse primer 5′-TCCTTCCTTTTCGGTTAGAGC-3′ (specific to the terminator sequence of all five yeast strains, as described [33]). Figure S1 was labeled using Powerpoint software.

### 4.4. Yeast Larvicide Assays

The yeast larvicides were evaluated through assays that were performed as described [50] and which conformed to the WHO larvicide testing guidelines [51]. In these assays, 20 freshly hatched first-instar larvae were placed in 500 mL plastic cups with 50 mL of distilled water and 50 mg of dried inactivated control-interfering RNA yeast [26] or dried inactivated yeast corresponding to *GGT*. The adult emergence rates and sexes of treated mosquitoes were recorded. Data compiled from four replicate trials were evaluated using the chi-squared test to identify statistically significant differences between the observed and expected 1 female: 1 male ratios, female survival, and male survival. The 1 female: 1 male ratio was established on the basis of extensive evaluation with control-interfering RNA yeast in each mosquito species evaluated.

### 4.5. Verification of Target Gene Silencing

Target gene silencing was verified through in situ hybridization assays that were executed on L4 larval brains as described [52]. Riboprobe corresponding to *GGT (AAEL017331)* was synthesized as specified [53] and used in these assays. Four biological replicate experiments were conducted on larvae (20 per replicate) that were treated with GGT yeast larvicides, as detailed above. After processing, larval brain tissue was mounted, viewed on a Zeiss Axioimager (Carl Zeiss Microscopy, LLC, Thornwood, NY, USA), and imaged using a Spot Flex camera (Diagnostic Instruments, Inc., Sterling Heights, MI, USA). Mean gray value analyses, which measure average signal intensity over the selected area, were conducted using FIJI ImageJ software [54], permitting the comparison of quantified digoxigenin-labeled transcript signals in the brains of larvicide treated vs. control larvae as described [55]. Data were statistically evaluated using Student’s *t*-test.

### 4.6. Scaled Rearing Trials

For scaled rearing of *A. aegypti,* 500 larvae were placed in 34 cm × 25 cm × 4 cm trays (1426B, Bioquip, Rancho Dominquez, CA, USA), which contained 1 L of distilled water. *A. aegypti* larvae were fed with the Zhang et al. mass-rearing diet [30], which consisted of a slurry containing 150 mg of ground shrimp (Tetra GMBH, Melle, Germany) and 250 mg bovine liver powder (MP Biomedicals, Santa Ana, CA, USA) mixed with 10 mL water, with the nutritional yeast diet component [30] replaced with dried GGT.546 yeast for sex separation treatments; larvae reared on the unmodified Zhang et al. [30] standard mass-rearing diet prepared with nutritional yeast served as a control. The nutritional yeast (control) or GGT.546 larvicidal yeast-interfering RNA component was combined with the shrimp-liver powder slurry and provided to larvae as follows: 100 mg at L1, L2; 200 mg at L3 and L4. For scaled rearing of *Culex* larvae, *C. quinquefasciatus* and *C. pipiens* were reared on a diet consisting of Koi food (Doctors Foster and Smith Koi Food, Rhinelander, WI, USA) mixed with either nutritional yeast (control) or larvicidal GGT.566 yeast and combined with 10 mL of water that was provided to the larvae as a slurry. For rearing one tray of 200 larvae, the amounts of Koi food and yeast combined with water and fed during each larval instar were as follows: L1: 120 mg Koi food + 40 mg yeast; L2: 120 mg Koi food + 40 mg yeast; L3: 160 mg Koi Food + 160 mg yeast; L4: 160 mg Koi Food + 160 mg yeast.

For all species, the larval rearing trays were examined daily for pupae, which were subsequently removed, sorted by sex, and counted. Mortality and sex ratio data for each control or larvicide treatment were evaluated in three replicate trials. Statistically significant differences between the observed and expected 1 male: 1 female survival ratios were evaluated using the chi-squared test, and the fitness of surviving adult males was estimated through comparisons of wing lengths, which correlate with fitness and were measured as described [55] and evaluated with Student’s *t*-test.

## 5. Conclusions

This investigation has uncovered a conserved female-specific requirement for *GGT* in distantly related disease vector mosquitoes. The results in conjunction with other recent functional analyses of genes in the sex-determining region of *A. aegypti* [21,22], as well as the orthologs of these genes in other species of mosquitoes [22], may have important implications for the study of mosquito sex chromosome evolution. Moreover, female-specific yeast-interfering RNA technologies that exploit the conserved female-specific requirement for *GGT,* as well as other M/m locus region genes and their orthologs in other mosquitoes, could support production of adult males. The use of other microbial systems could also be trialed for sex separation in *Culex* larvae. For example, *Pichia pastoris* [56] and *Escherichia coli* [57,58], which have also proven useful for expression and delivery of interfering RNA to mosquito larvae, could likely be used for silencing mosquito *GGT* genes and can be further assessed in *C. pipiens* complex mosquitoes. Moreover, given that the use of microbial systems for sex separation would require large-scale microbe production, it would be useful to pilot and optimize the scaled production of different microbes with the goal of determining which microbial-interfering RNA expression systems are the most economical and suited for scaled production. Such advancements would facilitate emerging population-based mosquito control strategies, particularly for species of the *C. pipiens* complex and other relatively less well-characterized mosquito species for which sex separation technologies are lacking.

## Figures and Tables

**Table 1:** Female-specific siRNA target sequences/genes and male:female sex ratios resulting from treatments.

siRNA	Target Sequence	Corresponding Genes	Species	siRNA Soaking Treatment #Males:#Females (N)	Male Mortality *p* Value	Female Mortality *p* Value
Control	GAAGAGCACUGAUAGAUGUUAGCGU	N/A	*A. aegypti*	20:20 (40)	1.00	1.00
546 ^1^	GAAGCAUUCAAACAUGCUUACGGCA	*AAEL017331*	*A. aegypti*	23:5 (40)	1.00	3.85 × 10^−7^
Control	GAAGAGCACUGAUAGAUGUUAGCGU	N/A	*A. albopictus*	20:19 (40)	1.00	1.00
565	GCAUCAAGCUUGAUGAUGAAAUUUA	*LOC109416314*	*A. albopictus*	22:5 (40)	0.48	4.93 × 10^−6^
Control	GAAGAGCACUGAUAGAUGUUAGCGU	N/A	*C. quinquefasciatus*	19:21 (40)	1.00	1.00
566	AGACUUACGAUUGAUGAAAACAGUA	*CPIJ016229*	*C. quinquefasciatus*	19:4 (40)	0.50	7.7 × 10^−8^
Control	GAAGAGCACUGAUAGAUGUUAGCGU	N/A	*A. gambiae*	19:18 (40)	1.00	1.00
560	CUAUCUGUUUGACCAGUUUACCGTC	*AGAP000853*	*A. gambiae*	19:3 (40)	0.25	1.28 × 10^−8^

^1^ siRNAs and corresponding target sequences/genes in the indicated species and the altered adult surviving male:female ratios resulting from siRNA soaking treatments are indicated. The *p* values displayed correspond to Fisher’s exact tests that compared observed and expected male or female survival (assuming 20 female: 20 male adult survivors, as predicted based on experimentation with these strains in our laboratory).

## Data Availability

All data are provided within the text.

## Supplementary Materials

The following is available online at https://www.mdpi.com/article/10.3390/pathogens11020169/s1, Figure S1: Expression of control, GGT.560, GGT.565, and GGT.566 shRNA was confirmed through PCR reactions, which produced ~100 bp amplicons from cDNA template prepared from total RNA isolated from each corresponding yeast strain. Results from two separate cDNA preparations (labeled 1 and 2 for each strain) are shown. No bands were produced in reactions that lacked cDNA template, which are marked as (-). The GGT.560, GGT.565, and GGT.566 primer sets failed to amplify bands from the control-interfering RNA yeast cDNA preparations (labeled cntrl 1 and 2). A DNA standard ladder is included in the far left and far right lanes.
