# Supplementary material for: A Conserved Female-Specific Requirement for the GGT Gene in Mosquito Larvae Facilitates RNAi-Mediated Sex Separation in Multiple Species of Disease Vector Mosquitoes
Source: Pathogens. 2022 Jan 27;11(2):169. doi: 10.3390/pathogens11020169 (PMC8879970; doi:10.3390/pathogens11020169)
Supplement: Supplementary file 1 [file pathogens-11-00169-s001.zip › pathogens-1525374-supplementary.pdf]

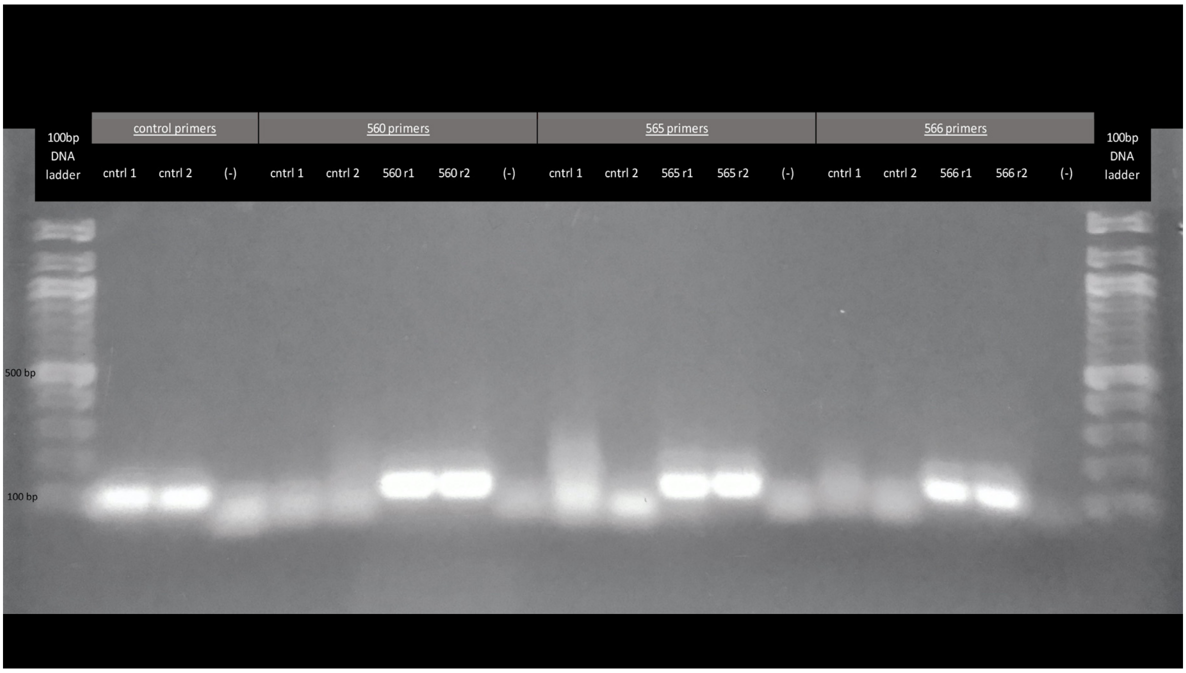

Figure S1. Expression of control, GGT.560, GGT.565, and GGT.566 shRNA was confirmed through PCR reactions, which produced ~100 bp amplicons from cDNA template prepared from total RNA isolated from each corresponding yeast strain. Results from two separate cDNA preparations (labeled 1 and 2 for each strain) are shown. No bands were produced in reactions that lacked cDNA template, which are marked as (-). The GGT.560, GGT.565, and GGT.566 primer sets failed to amplify bands from the control-interfering RNA yeast cDNA preparations (labeled cntrl 1 and 2). A DNA standard ladder is included in the far left and far right lanes.
